# The Global Burden of Road Injury: Its Relevance to the Emergency Physician

**DOI:** 10.1155/2014/139219

**Published:** 2014-01-21

**Authors:** Sharon Chekijian, Melinda Paul, Vanessa P. Kohl, David M. Walker, Anthony J. Tomassoni, David C. Cone, Federico E. Vaca

**Affiliations:** ^1^Department of Emergency Medicine, Yale University School of Medicine, 464 Congress Avenue, Suite 260, New Haven, CT 06519, USA; ^2^Yale University, New Haven, CT 06520, USA; ^3^Department of Family Medicine, White Memorial Medical Center, Los Angeles, CA 90033, USA; ^4^Division of Pediatric Emergency Medicine, Elmhurst Hospital Center, Ichahn School of Medicine at Mount Sinai School, Elmhurst, NY 11373, USA

## Abstract

*Background*. Road traffic crash fatalities in the United States are at the lowest level since 1950. The reduction in crash injury burden is attributed to several factors: public education and prevention programs, traffic safety policies and enforcement, improvements in vehicle design, and prehospital services coupled with emergency and acute trauma care. Globally, the disease burden of road traffic injuries is rising. In 1990, road traffic injuries ranked ninth in the ten leading causes of the global burden of disease. By 2030, estimates show that road traffic injuries will be the fifth leading causes of death in the world. Historically, emergency medicine has played a pivotal role in contributing to the success of the local, regional, and national traffic safety activities focused on crash and injury prevention. *Objective*. We report on the projected trend of the global burden of road traffic injuries and fatalities and describe ongoing global initiatives to reduce road traffic morbidity and mortality. *Discussion*. We present key domains where emergency medicine can contribute through international collaboration to address global road traffic-related morbidity and mortality. *Conclusion*. International collaborative programs and research offer important opportunities for emergency medicine physicians to make a meaningful impact on the global burden of disease.

## 1. Introduction

Deaths caused by road traffic injuries (RTI) in the United States are at their lowest number since 1950, with a nearly 10 percent decline in such fatalities between 2000 and 2009 alone [1]. Though the National Highway Traffic Safety Administration (NHSTA) cites several factors in the reduction of road traffic fatalities, including economy, unemployment, and improvements in vehicle design, success can be attributed to the attention focused on prehospital services and emergency medicine (EM) in the last half century, as well as the attention paid to injury prevention efforts within the field of EM itself.

The passage of the 1966 Traffic and Motor Vehicle Safety and Highway Safety Acts set unprecedented federal safety standards for motor vehicles and state highway safety programs [2]. In establishing such standards at federal and state levels, road safety compliance became a greater and more tangible priority. The next few decades saw an increase in federal funding, which was applied toward mass media awareness campaigns and research grants, as well as driving while intoxicated (DWI) regulations, and seatbelt and drinking-age laws [2].

With the increase in federal funding and the sense of urgency created by the 1966 publication, *Accidental Death and Disability: The Neglected Disease of Modern Society*, came the creation of emergency medical services (EMS) in the 1960s and 1970s [3]. EM, a newly recognized specialty, saw the need to bring advances to the pre-hospital care arena in cardiac and resuscitation interventions and in trauma care, helping to promote and strengthen the growth in EMS systems.

In the subsequent years following the establishment of an organized EMS system, a significant decrease was observed in RTI fatalities that would continue as an overall decrease in the decades to follow [4]. By applying scientific and research-based methods towards traffic safety aims, the United States was able to achieve great success in reducing RTIs [5]. Legislative efforts, policy making, engineering and medical advances, research initiatives, and the overall cooperation of various sectors in the United States all contributed to creating safer roads, an accomplishment so significant that it was recently recognized by the Centers for Disease Control and Prevention (CDC) as one of the top ten great public health achievements in the last decade [6].

In contrast to favorable downward trends in the US, the global burden of disease attributed to RTIs has continued to increase. In this report, we describe this worrisome trend by highlighting the escalating global disease burden of RTIs and examining global initiatives focused on RTI and fatality reduction. We conclude by identifying and suggesting four key domains where EM can make a meaningful impact with its international partners to reduce this global burden of disease.

## 2. Review

### 2.1. The Global Burden of Injury

The global burden of RTIs continues to grow and promises to overtake tropical diseases as a leading cause of death in the developing world. In 1990, RTIs ranked ninth in the ten leading causes of the global burden of disease, with respiratory infections and varying diseases in the top rankings [7]. By 2030, however, it is projected that RTIs will rank fifth in leading causes of death.

In 2004, the World Health Organization (WHO) and the World Bank combined efforts to publish the *World Report on Road Traffic Injury Prevention*, a result of a decade long discussion, to provide insight into the growing public health, economic, and social burdens caused by RTIs [5]. The report projected a rise in RTIs and fatalities, with an overwhelming and disproportionate 80% increase in low- and middle-income countries (LMIC). With an annual death toll of approximately 1.3 million people worldwide, the recent worsening of RTI statistics, and the dismal projections for the next two decades, failing to take significant, immediate action will lead to devastatingly increased death rates in an avoidable and preventable global health epidemic.

One factor in the rise of RTIs is the undeniable link between increased road travel and economic growth and prosperity. Despite the necessity of travel to fuel the economy, the weight of the monetary burdens created by RTIs is increasing [5]. According to the 2009 *Global Status Report on Road Safety*, RTIs have yielded an estimated US$518 billion in global losses, costing governments between 1% and 3% of their gross national product (GNP) [4]. This finding is disproportionally significant in low-income countries, where economic costs of RTIs exceed developmental assistance. The burden lies on national and local economies but also on individual households [5]. While mortality due to RTIs continues to decrease in higher-income countries, the projected increase in road traffic fatalities in low-income countries places monetary burdens on the global economy affecting high-, middle-, and low-income countries alike.

The social impact of global RTIs also continues to become a more tangible burden. Alongside economic costs, road traffic fatalities contribute to public health concerns, particularly among the world's younger populations. Between the ages of 5 and 44, RTIs are in the top three causes of death [5]. Not only does this affect the number of disability-adjusted life years lost (a measure combining years lost in premature death with health loss due to disability), but it also creates an added burden to families in low-income countries where the main source of household income is often from the manual labor of young hands [8]. In this way, RTIs decrease productivity and increase health costs, proving detrimental to already struggling households and economies.

Without global action, the outlook for the next few decades is far from promising. It is estimated that, despite a decrease of 30% in deaths related to RTIs in high-income countries, road traffic fatalities will continue to increase dramatically in low- and middle-income countries [5]. With such high costs for such a preventable global problem, the world has taken notice. Still, considerably more tangible day-to-day action needs to be taken.

### 2.2. Major International Initiatives from Development Agencies

The international development community has taken notice of this burgeoning toll of unintentional injury. The WHO and the World Bank, along with over 100 experts from around the world, ignited the discussion on global road safety through the 2004 *World Report on Road Traffic Injury Prevention* [5]. Their aim was to encourage governmental and societal attention to a growing burden and to fuel immediate action in the fight to decrease RTIs by presenting the facts about road traffic safety. With the *World Report on Road Traffic Injury Prevention*, the WHO and the World Bank sparked a cascade of international initiatives and campaigns toward safer roads.

The Pan American Health Organization (PAHO) organized the Pan American Conference on Road Safety just after the publication of the *World Report on Road Traffic Injury Prevention*. The conference united 16 countries in conversation in 2005 [9]. PAHO is particularly targeted toward improving public health in the Americas, working as part of the United Nations to focus especially on underresourced, more vulnerable populations. Citing the importance of consolidating efforts between countries to reduce the number of RTIs, PAHO sought to develop a pan-American road safety network to promote the adoption of the recommendations given in the *World Report on Road Traffic Injury Prevention*.

The World Bank's Global Road Safety Facility also expanded on the *World Report on Road Traffic Injury Prevention* by developing broader initiatives for combating the growing threat of RTIs [10]. Launched in 2005, its mission is to provide seed funding for the implementation of road safety programs in low- and middle-income countries.

Prior to the publication of the *World Report on Road Traffic Injury Prevention*, the United Nations General Assembly called on the WHO to coordinate the initiative to address road safety issues within the UN system [11]. Upon this appointment, the WHO established the United Nations Road Safety Collaboration (UNRSC), which initiated work in coordinating efforts between international participants in the global road safety network, developing manuals and databases and various other organizing tools to help public agencies develop interventions.

### 2.3. International Programs, Associations, and Safety Campaigns

The growing road safety crisis has been met by increased collaboration between international governments and agencies. The Global Road Safety Partnership particularly addresses the governments of LMIC, aiming to intervene in the collaboration of societal sectors (business, civil, government, etc.) at all levels (global, national, and local) to create a focus for road safety efforts [12]. The Partnership focuses on specific countries, including the Association of Southeast Asian Nations (ASEAN), Brazil, China, Hungary, India, Namibia, Poland, Romania, the Russian Federation, and South Africa, that fit particular criteria based on need and level of governmental political will to address the problem. The Bloomberg Philanthropies has also joined this important cause with the Global Road Safety Initiative. Bloomberg is targeting the ten LMICs that account for half of RTIs worldwide: Brazil, Cambodia, China, Egypt, India, Kenya, Mexico, Russia, Turkey, and Vietnam.

Alongside the Global Road Safety Partnership, the International Road Safety Academy (IRSA) Association also aims to provide support to LMICs in lowering the number of RTIs [13]. Initiated in 2004 by the Institute of Traffic Care and part of the European Road Safety Charter, its focus is encouraging improved road safety education and training, along with technological advances, to better equip future policy makers and agencies.

The International Road Federation, a not-for-profit, non-governmental organization founded in 1948, seeks to combine the efforts of commercial and professional firms, corporations, public and governmental organizations, educational and research institutions, and national and regional road associations like the IRSA [14]. It was first established to promote the development of efficient highway systems after World War II [15]. Since then, it has aimed to improve the safety of road networks worldwide by creating a forum on road safety from a developmental and maintenance-oriented standpoint [14].

Representing the United States in this global forum, the Association for Safe International Road Travel is a nonprofit, international road safety organization that seeks to encourage safe roads through advocacy and education [16]. Founded in 1995 and still the only such organization in the United States, it encourages safer travel by providing and annually updating road travel reports for over 150 countries. It works with WHO, the UN, and the World Bank to foster conversations on international travel safety.

The Global Road Safety Forum (GRSF), founded in 2002 and part of the Task Force for Global Health, provides support for regional and global international road safety initiatives that stem from such conversations [17]. Regionally, the GRSF is conducting road safety projects in Latin America, the Caribbean, and South Asia. Internationally, the GRSF also holds forums on global road safety, encouraging open discussion of safety initiatives to raise awareness of the global burden resulting from RTIs.

One such awareness-raising initiative is the Make Roads Safe campaign, which began in 2006 in response to the *World Report on Road Traffic Injury Prevention* and in coordination with the Fédération Internationale de l'Automobile (FIA) Foundation [18]. The campaign's major initiative was the proposal of the “Decade of Action for Road Safety,” declared by the United Nations General Assembly in 2010 as the years from 2011 to 2020 [19]. The goal in declaring this decade of action was to encourage cooperation between UN member nations at the global, national, and regional levels to decrease the number of road traffic fatalities by five million worldwide. The “Decade of Action” was declared immediately following the First Global Ministerial Conference on Road Safety, adopted by the UN General Assembly and held in 2009.

### 2.4. Significance and Opportunities for Emergency Medicine

The recently observed trends in global RTIs and fatalities demonstrate that current approaches to global road safety are falling short. A comprehensive injury prevention and control model is better suited to the task. To encourage action at all levels, it must be directed by those locally with influence within those same spheres. Emergency physicians have a unique opportunity to directly influence this field, as they are already participating in locally and nationally based education, EMS, acute care, and research initiatives, policymaking, and advocacy relevant to road traffic safety. Emergency physicians have learned many important lessons from the injury prevention and control activities in which they are engaged. It is therefore important that emergency physicians from those countries with longstanding experience broadly participate in assisting organizations and countries committed to global road safety and reducing the burden of injury. In turn, this promises to improve global health. Throughout their own development and evolution, US and European EM have been notably successful in making impactful contributions to national traffic safety [20]. Much of this success stems from the development and perpetual refinement of EMS, injury prevention and control, research, and policy and advocacy efforts. Each of these domains with their history of domestic success offers a highly relevant and ripe opportunity for translation to the global road safety context. Ultimately the commitment and political will must come from local physicians. We should be ready to lend support to them where it is needed in their endeavors. Without the clinical dimension and input from local physicians, the efforts of the development community will fall short. The policies need “feet on the ground” to be enacted but they also need to be informed by those who work on the frontlines. It is those who are impassioned by prevention of RTIs and trauma care in their respective countries that will ultimately make the biggest impact on reduction of traffic related morbidity and mortality. It is the synergy between already honed expertise and local political will that will serve to reduce this public health threat on a global scale.

The opportunities for collaboration are myriad. Areas with high impact potential are EMS system development, injury prevention control measures, injury prevention research, and policy and advocacy work.

### 2.5. EMS System Development

Emergency medical services (EMS) refer to the network of dispatch, ambulances, and receiving hospitals that combine to provide care for injured and ill patients. EMS system development and organization must be appropriate to the setting in order to make efficient use of existing scarce human, material, and financial resources and to facilitate future resource development and training of providers to best address global road safety. In the past, global health policy has focused on a disease specific model with interventions targeting each disease. Little attention was paid to the development of a comprehensive emergency medical system [21]. As a result, there is a great disparity among the EMS capacities of varying nations and their regions. Local system planners should reflect on best practices and components of a well-developed system within local constraints. The degree of EMS development will depend largely on available funding. In resource poor areas, we should look towards incremental progress towards the best practices.

Pre-hospital care worldwide can be organized into four categories, “unorganized pre-hospital care” where lay people or police transport patients to the hospital, basic life support systems where EMTs provide noninvasive care and transport, advanced life support systems that are staffed by highly trained paramedics that use protocols to drive their care, and finally Doc-ALS systems where physicians deliver pre-hospital care [22].

In regions that have addressed the development of EMS systems worldwide, there have been two main directions taken, the Anglo-American model based on a “scoop and run” system mainly staffed by paramedics and the Franco-German model largely staffed by physicians. In the Franco-German model, patients are treated on site and only transported to the hospital if they require further care [22]. In each particular country, the type of system that has been adopted is of utmost importance when formulating a national or regional strategy in RTI prevention. It is essential that emergency physicians worldwide take part in the dialogue that will form both the EMS and the policy backbone. They alone know the intricacies of the way their EMS systems have developed or need to develop in order to address and minimize injury morbidity and mortality.

In regions where EMS systems have yet to be developed the preference for “scoop and run” practices rather than “stay and play” should be stressed. In underresourced countries the goal should be to decrease the time to definitive care and to channel energies towards the development of trauma centers. There has been little scientific support given to increasing advanced medical care given on scene in a trauma response. In fact, if specific pre-hospital interventions such as advanced airway management, intravenous fluid administration and immobilization and splinting are examined, only immobilization and splinting are shown to be beneficial especially in areas with shorter transport times such as urban areas. In more rural areas with longer transport time, more advanced interventions may be warranted if those in the field are properly trained or have advanced training such is the case when physicians respond to calls [23]. Several studies including the OPALS study from 2008 have compared basic life support (BLS) to advanced life support (ALS) and concluded that there were no outcome improvements in morbidity or mortality [24]. Other meta-analyses have actually shown adverse outcomes in severity-matched cases in those transported by ALS units versus BLS units [25]. All of these findings must be interpreted in relation to the quality of both the EMS system and the level of training of its providers. Given these constraints, even in developed EMS systems such as Canada's, little advantage can be gained from a “stay and play” approach in traumatic injuries.

There are innumerable instances where well-meaning philanthropists have donated new, well-equipped, western style ambulances to rural areas of LMICs in areas that have no communication infrastructure to manage use of the ambulance, no trained personnel to staff it, and no medical infrastructure to accept patients that the ambulance might transport [26]. Local needs must be defined and prioritized to support the work of the doctors, community health workers, advocates and legislators. By becoming involved in the earlier stages of EMS development, local physicians can ensure that the needs of their patients are met and that their efforts are supported. Efforts are underway to define EMS needs and shortcomings at a local level. Initial needs assessment work by Razzak et al. in Pakistan found that 98% of community survey respondents were dissatisfied with the currently provided emergency medical care. Ninety-eight percent of providers felt that their facilities were not equipped to provide emergency care [27]. Further system funding, development, and expertise are sorely needed.

Many organizations offer resources to aid in the development and evolution of EMS systems: EMS, EM, and trauma professional organizations worldwide, such as ACS and NAEMT, WHO, PAHO, the UN Road Safety Organization, and the US Department of Transportation. Opportunities for interested parties to become involved in enhancing international EMS development are plentiful and include opportunities to engage in planning, policy development, teaching, oversight, and direction. In order for this collaboration to take place, local partners must first find the drive to address and ameliorate these worthy concerns. Once they do so, the international community must be ready to lend its expertise.

In many areas of the world, EM does not yet exist as a formalized specialty or the specialty is in its infancy. The development of EM is essential in the efficient and accurate delivery of care for trauma patients as well as in medical direction for the overall EMS system. In areas where the specialty of EM does not exist, there are often early proponents of the specialty from diverse backgrounds such as cardiology, internal medicine, and general surgery. Very often these early proponents either seek additional training for themselves or for the next generation of physicians entering the nascent specialty. The development of the specialty and the development of emergency medicine residency training can benefit from the collaborative expertise of more experienced partners and educators who have already advanced the specialty in their own countries and regions.

Systematic training according to ATLS training programs and guidelines ensures a protocol driven approach to complex traumatic issues and allows physicians from diverse specialties such as surgery and orthopedics to approach injuries with a consistent and comprehensive framework. These training modules devised by the American College of Surgeons (ACS) have been shown to mitigate global road fatalities, injuries, and long-term disabilities. These ACS modules include advanced trauma and life support (ATLS) [28], (http://www.facs.org/trauma/atls/), Advanced traumatic operative management (ATOM) [29], (http://www.facs.org/trauma/atom/), and Advanced Surgical Skills for Exposure in Trauma (ASSET) [30], (http://www.facs.org/trauma/education/asset.html). Moreover, the ACS has devised a strategy for the international dissemination of its educational models with over sixty countries participating voluntarily [31], (http://www.facs.org/trauma/atls/promulgation.html/). In addition, the National Association of Emergency Medical Technicians has partnered with the ACS to develop global continuing medical education for pre-hospital providers with the pre-hospital trauma and life support program or PHTLS [32], (http://www.naemt.org/education/PHTLS/whatisPHTLS.aspx).

There are many examples of recent collaborations that illustrate the efforts already undertaken in EMS development. The Department of Emergency Medicine at Emory has had significant involvement over the last several years in the area of EMS development in Ghana, Mozambique, and Georgia. Their involvement in Tbilisi, Georgia, funded by USAID, is focused on EM specialty and residency development in an area where a group of like-minded local doctors had decided to establish the first emergency department in ex-Soviet Georgia [33]. Their efforts to date have resulted in the development of a “miniresidency” certificate program that is taught by local faculty with support from the faculty at Emory.

### 2.6. Injury Prevention and Control Measures

While the field of injury prevention in low- and middle-income countries is immature compared to that in the United States, there are lessons learned in the US and other developed nations that can be effective in promoting a culture of road safety in these regions. Through a network of likeminded partners, emergency physicians and their trauma colleagues can be the vehicles through which successful programs can be adapted and implemented in other parts of the world, and there are a variety of ways in which they can promote and become involved in efforts to control injuries from road crashes (Table 1).

The WHO, in its 2004 *World Report on Road Traffic Injury Prevention*, outlines several key points that should frame any discussion on injury prevention efforts (Table 2).

While there is no “one standard approach” to injury prevention, interventions that are most effective are those encompassing a variety of strategies, including legislation, environmental modification, and public education. Interventions should reach across different sectors, build on existing networks, and involve communities. They need to be tailored to the social and physical environments of the target locales.

An example of such an intersection of multiple sectors exists in Thailand where recent attention has been paid to motorcycle crashes. Researchers have collected data to characterize the nature of motorcycle accidents, to understand the effectiveness of helmets, and to elucidate the effect of legislation on the use of motorcycle helmets. Recent efforts have culminated in a declaration by the government of Thailand that 100% crash helmet use will be targeted over the course of 2011. This federal program relies on the government's Departments of Education and Labour to educate the public and promote helmet use for all workers. It also relies on the police force for improved enforcement of a mandatory helmet law. Another example of this type of enforcement intervention can be found in work being done by the author in the Republic of Armenia in conjunction with the Department of Emergency Medicine at Yale. Car safety device usage was examined in a baseline study. An enforcement intervention followed and a second study was performed to document whether there was an improved awareness and usage in the study population. The follow-up study showed an overwhelming improvement in both areas after intervention [21].

### 2.7. Injury Prevention Research

While interventions are the mainstay of reducing traffic-related morbidity and mortality, quality research is needed to guide policy, programs, campaigns, and interventions. Substantial work is needed on research capacity development, particularly in the developing world where a paucity of trained researchers, funding, and research infrastructure persist [5]. While a number of national road traffic crash research centers exist and have made substantial progress in addressing road traffic safety, most are in the high-income countries, with national centers in Holland, Germany, and England, and regional centers in North Carolina and Michigan in the US and South Australia and Victoria in Australia. Notable exceptions are the Institute of Technology in New Delhi, India, and the Centre for Industrial and Scientific Research in South Africa, both of which have made substantial contributions to understanding road injury problems in LMICs [5].

To date, outside of these established research centers, there are few studies of a rigorous nature performed in LMICs, where the burden of RTIs is most concerning. Most articles within this body of knowledge are descriptive or simply quantify the burden of RTI in various locations [21]. Likely this is due to a lack of funding for more substantial research efforts. Little is known about the science of road injury prevention or pre-hospital treatments and interventions in LMICs. Nor do we understand attitudes and beliefs surrounding safety device usage in their populations. Attention needs to be paid to all links in trauma care such as emergency department care, surgical intervention when needed, diagnostic imaging with limited resources, and rehabilitation. Increased research support both in terms of capacity building and in funding for such research needs to be directed towards LMICs. It is the duty of EM physicians to prioritize the importance of this research to their mission to the other stakeholders and policy makers within governmental and nongovernmental organizations. It is not enough to devise a strategy. The strategy needs to be informed by useful data.

In many parts of the world where trauma care systems are underdeveloped and resources are limited, even basic descriptive epidemiological information and data are lacking. While many nations have some form of surveillance system in place for collecting road crash data, both the quality and reliability of these data vary widely, often within, as well as between, nations [34]. The development of trauma registries is of the utmost importance in strengthening the mission of road injury prevention and to allow for the development and implementation of local and regional data-driven prevention activities and efforts. Before the development of any injury prevention initiative, it is important to understand the nature and consequences of injury among the population to be targeted. Prevalence and incidence data are needed to properly and accurately inform and guide prevention activities and efforts. Disease and injury surveillance systems are indispensible in addressing the global burden of injury morbidity and mortality.

Without reliable data, it is nearly impossible to decide how to prioritize and develop effective programs. Furthermore, it is also difficult to convince local leaders, policy makers, and others that an injury problem exists and that it truly threatens the health of the entire community. Where resources are limited, it is important to collect enough data to make analysis clear and demonstrative. It is equally important to not to collect so much data that it becomes expensive and cumbersome. In order for the data to be useful and complete, a decision regarding which regions and hospitals should be included and whether or not pre-hospital systems should populate the registry needs to be made by all stakeholders including government agencies [35].

The goal of injury surveillance is to analyze modifiable risk factors and to understand populations at higher risk of injuries from road crashes. The establishment of data collection systems and hospital surveillance, development of community-based surveys, and initiation of other appropriate research can assist in evaluating the scope, characteristics, and consequences of crashes. Both the establishment of regional road crash databases (such as the Asia-Pacific Road Accident Database) and the development of international guidelines on core minimum data sets and supplementary information to be collected for all victims of injury (including road traffic victims) should help improve the quality and reliability of such surveillance data [36].

These trauma registries may exist in different stages around the world. In most LMICs, we can expect that they do not yet exist at all. The role of the local physician in the development of these registries cannot be underestimated. It takes a firm commitment to the process of collecting, entering, and maintaining records for the registry. It also takes clinical knowledge to populate the registry with accurate and pertinent information that can act as a supporting data to the mission at hand. Collaborators from regions with more experience in developing and maintaining registries can provide a shortcut for those just beginning to establish theirs. Their experience can save much frustration and unnecessary duplication of efforts.

The Queen Elizabeth Central Hospital in Blantyre, Malawi, in collaboration with trauma experts at Yale University School of Medicine, instituted a trauma registry to track and analyze all injuries for pediatric patients. This system will allow the hospital to report data that will inform future injury prevention and quality improvement efforts. Additionally, the data will be used to advocate for a national trauma registry and increased attention toward traffic injuries as a health priority. This program's success lies in its partnering of two academic centers, technology transfer via use of specialized trauma database software, and transfer of lessons learned from the management of a trauma registry at a major US trauma center.

Other efforts are underway in Mozambique in collaboration with Emory's Department of Emergency Medicine. The project has focused on training public health officials the concepts of trauma care as well and injury prevention control methods. This course was used as an impetus to start a trauma registry and laid the groundwork for future injury prevention research as well as the development of an “injury control research center.”

The Fogarty International Center has been active in funding programs in injury prevention research development. In 2006, the center funded the International Research Training Program in Trauma and Injury Prevention in Colombia in collaboration with the University of Texas in order to build capacity for injury prevention research. The stated goals of the project are to “train foreign scientists and professionals in trauma and injury surveillance, prevention, control, and evaluation through an academic training and research program” [37, 38]. Felknor et al. have highlighted the importance of funding pilot projects in LMICs as a means of practical training of future injury prevention researchers in country [39].

### 2.8. Policy and Advocacy

Advocacy by stakeholders is vital to the development of policy. Emergency physicians are in a unique position to understand and therefore to advise on matters pertaining to global traffic safety, as a result of both clinical exposure and training in running and giving medical direction to EMS systems. Within the community, they are the eyes and ears of the politicians as roads, bridges, and pedestrian walkways are planned and implemented. Locally, emergency physicians, along with EMS personnel, know the most dangerous intersections and interchanges in their communities. Globally they understand the importance of systemic changes that can aim to eliminate the undue burden that road traffic injuries levy on the youngest and most productive members of society.

Areas of opportunity for effective global policy include legislation governing driving while intoxicated, traffic speed limits, mandated safety device presence, and usage in cars and two-wheeled vehicles, including child safety seats, seatbelts, and helmets. Also important is careful work with road safety engineers to eliminate hazards introduced by roads and road conditions and development and maturation of EMS systems.

A critical component to the effects of policy will continue to be enforcement. Without credible and meaningful enforcement, policies that are in place will not have the desired effect. Assurance that traffic safety policy will be effective is largely predicated on the careful coordination of the matrix between community groups, politicians, non-governmental organizations, road safety engineers, and automotive experts, with the emergency physician at the center.

An important example of collaboration in the area of policy and advocacy is the Fogarty funded “International Collaborative Trauma and Injury Research Training Grant” awarded to Johns Hopkins and Aga Khan University in Pakistan. The stated goals of the project are to “focus on using US expertise to strengthen Pakistani institutions, promote a sustainable research enterprise focused on acute care of trauma and injuries, and enable national dissemination of research evidence to influence policy and investments in Pakistan and its future.” The project aims to develop trauma and injury research expertise at a public sector institution in Pakistan. This research will focus on providing data to support and inform key national priorities for trauma care and injury prevention. This will result in an increased capacity to sway policy and enact interventions based on a measurable impact. This project is a perfect illustration of a motivated group from a LMIC who is collaborating with a partner with the desired expertise in order to prioritize and realize their own national goals and agenda [38]. Stemming from this project, Waseem et al. describe the epidemiology of crashes in Punjab, Pakistan, and use their findings to point out areas for improvement in policy, legislation, and enforcement, using their pilot work to inform the national injury prevention agenda [40].

These examples highlight successful, multisectorial, multidisciplinary programs aimed at prevention of injuries from traffic accidents. EM providers can be involved in the pre-hospital setting, the hospital/emergency department level, or by developing programs focused on increasing access to emergency care and rehabilitation.

Advocacy on behalf of patients can take many forms. It can take persistence to ensure the establishment, updating, and optimization of EMS around the globe in order to take care of patients in their time of need. It can mean advocating for injury prevention measures and insisting that adequate resources are allocated toward enforcement. It can mean working with lawmakers and law enforcement groups to help craft culturally appropriate legislation. It can mean working with communities around the world to stress the importance of safety device usage and to ensure that they become culturally accepted and relevant. In areas where overcrowded buses or overburdened two-wheeled vehicles are employed, it can mean assisting in the long-term planning and prioritization of safe public transportation systems. As the global population grows and becomes increasingly dependent on motorized transportation, emergency physicians will have to work hard to eliminate its ill effects. Advocacy means going above and beyond daily duties as doctors to ensure that politicians know about the growing burden of disease inflicted by these inevitable changes.

## 3. Conclusions

By working on the implementation of road and traffic injury prevention measures both at home and abroad, emergency physicians can share expertise, experiences, and successes in road injury prevention, reducing the global impact of road traffic injuries. Important opportunities for transfer and translation of national successes in road injury prevention through EMS development, injury prevention research, intervention, policy, and advocacy are essential to the mission. Identification of key stakeholders in LMICs combined with intentional cooperation of international emergency physicians, global health organizations, and legislative as well as policymaking sectors can significantly strengthen the road traffic safety initiative worldwide and will result in decreased morbidity and mortality attributed to traffic related injury.

## Figures and Tables

**Table 1:** Ways for EM practitioners to get involved in global road safety.

Data collection. Initiate or assist with improved injury surveillance	

Advocacy. Working to persuade decision-makers to address injuries as a major issue and of the importance of adopting improved approaches to road traffic safety	

Programming. Designing, implementing, monitoring, and evaluating appropriate interventions	

Capacity building. Promoting capacity of health care systems to recognize and care for and provide rehabilitation to victims of road traffic injuries	

Knowledge transfer. Adapt successful initiatives from the developed world to low resource settings	

Partnering. Promote global collaboration by forming partnerships between academic institutions and foreign governments, health centers, or NGOs	

Education. Incorporate lessons on road traffic crashes into medical school and residency curricula; educate healthcare providers and patients of safe behaviors	

**Table 2:** Key points for global road traffic injury prevention (WHO, 2004) [5].

Road crash injury is largely preventable and predictable; it is a human-made problem amenable to rational analysis and countermeasure	

Road safety is a multisectorial issue and a public health issue—all sectors, including health, need to be fully engaged in responsibility, activity, and advocacy for road crash injury prevention	

Common driving errors and common pedestrian behavior should not lead to death and serious injury—the traffic system should help users to cope with increasingly demanding conditions	

The vulnerability of the human body should be a limiting design parameter for the traffic system and speed management is central	

Road crash injury is a social equity issue—equal protection to all road users should be targeted for since nonmotor vehicle users bear a disproportionate share of road injury and risk	

Technology transfer from high-income to low-income countries needs to fit local conditions and should address research-based local needs	

Local knowledge needs to inform the implementation of local solutions	
